# The Arts and Tools for Using Routine Health Data to Establish HIV High Burden Areas: The Pilot Case of KwaZulu-Natal South Africa

**DOI:** 10.3389/fpubh.2019.00335

**Published:** 2019-11-12

**Authors:** Njeri Wabiri, Inbarani Naidoo, Esther Mungai, Candice Samuel, Tryphinah Ngwenya

**Affiliations:** ^1^Social Aspects of Public Health Research, Human Sciences Research Council, Pretoria, South Africa; ^2^Kwa-Zulu Natal (KZN) Provincial Treasury Global Fund Supported Programme, Pietermaritzburg, South Africa; ^3^KZN Provincial Department of Health-GIS Directorate, Pietermaritzburg, South Africa

**Keywords:** routine health facility data, Africa, HIV, “Hotpots”, Big Data, spatial interpolation

## Abstract

**Background:** To optimally allocate limited health resources in responding to the HIV epidemic, South Africa has undertaken to generate local epidemiological profiles identifying high disease burden areas. Central to achieving this, is the need for readily available quality health data linked to both large and small geographic areas. South Africa has relied on national population-based surveys: the Household HIV Survey and the National Antenatal Sentinel HIV and Syphilis Prevalence Survey (ANC) amongst others for such data for informing policy decisions. However, these surveys are conducted approximately every 2 and 3 years creating a gap in data and evidence required for policy. At subnational levels, timely decisions are required with frequent course corrections in the interim. Routinely collected HIV testing data at public health facilities have the potential to provide this much needed information, as a proxy measure of HIV prevalence in the population, when survey data is not available. The South African District health information system (DHIS) contains aggregated routine health data from public health facilities which is used in this article.

**Methods:** Using spatial interpolation methods we combine three “types” of data: (1) 2015 gridded high-resolution population data, (2) age-structure data as defined in South Africa mid-year population estimates, 2015; and (3) georeferenced health facilities HIV-testing data from DHIS for individuals (15–49 years old) who tested in health care facilities in the district in 2015 to delineate high HIV disease burden areas using density surface of either HIV positivity and/or number of people living with HIV (PLHIV). For validation, we extracted interpolated values at the facility locations and compared with the real observed values calculating the residuals. Lower residuals means the Inverse Weighted Distance (IDW) interpolator provided reliable prediction at unknown locations. Results were adjusted to provincial published HIV estimates and aggregated to municipalities. Uncertainty measures map at municipalities is provided. Data on major cities and roads networks was only included for orientation and better visualization of the high burden areas.

**Results:** Results shows the HIV burden at local municipality level, with high disease burden in municipalities in eThekwini, iLembe and uMngundgudlovu; and around major cities and national routes.

**Conclusion:** The methods provide accurate estimates of the local HIV burden at the municipality level. Areas with high population density have high numbers of PLHIV. The analysis puts into the hand of decision makers a tool that they can use to generate evidence for HIV programming. The method allows decision makers to routinely update and use facility level data in understanding the local epidemic.

## Introduction

The HIV epidemic in South Africa is complex with diverse factors driving the epidemic regardless of spatial boundaries. The epidemic is also heterogeneously distributed in different geographic areas (1). The urgent need to better understand subnational variations in HIV epidemiology is key to programme planning. It is therefore important to conduct HIV epidemic appraisals across different geographic areas to help better characterize the drivers of the epidemic and ensure that HIV intervention programmes match the local epidemic context, with resources allocated to interventions that have the greatest impact locally. Central to achieving this, is the need for readily available quality health data linked to both large and small geographic areas. Over the years, South Africa has relied on national population based surveys such as the Household HIV Survey (2) and the National Antenatal Sentinel HIV and Syphilis Prevalence Survey (ANC) (3) amongst others, to provide data for informing policy decisions. However, these surveys are conducted approximately every 2 and 3 years creating a gap in data availability and evidence required for decisions at provincial, districts, and municipalities levels. At these administrative levels, timely decisions are required with frequent course corrections in the interim. Actions emanating from policy directives require information from various sources to be streamlined sometimes at a rapid pace to be used effectively (4). Hence, there is a need to use whatever health information is at hand, in the best possible way to inform such decisions. The South African District Health Information System (DHIS) contains aggregated routine health data from public health facilities, and can be used to close this gap. The DHIS was developed to collect aggregated routine data from all public health facilities, intended to support decentralized decision making and health service management (5). It is used in several other low and medium income countries (LMIC) (6). The DHIS data can be integrated with high resolution population data to, for example, generate estimates of HIV disease burden by estimating the number of PLHIV at any geographic level. Availability of such estimates at low geographic areas is a powerful tool for decision makers who need to prioritize allocation of limited health resources (4) and can also be used as case studies for ongoing epidemic monitoring. The study is a collaboration between the Human Sciences Research Council spatial analysts, the KwaZulu-Natal (KZN) Provincial Treasury Global Fund Supported Programme, and the KZN Department of health including decision makers at district and municipality levels.

The purpose of this study is to describe the methodology used to produce the estimates of HIV disease burden at a 100 m resolution to municipality and district level using routine facilities HIV testing data to support local decision making. We outline step-by-step approach for the decision makers to follow to produce estimates for guidance in decision making.

## Materials and Methods

### Data

The study combine three types of data.

#### Age-Structure Data

We obtained the age–structured data as defined in the South Africa Census 2011 and the Mid-year population estimates, 2015 from the Statistics South Africa (StaSSA) (7).

#### DHIS HIV Data for 15–49 Years Old Clients'

The DHIS HIV data describes quality checked totals for confirmed HIV tests at public health facilities. The data used in the study consist of a total of 887 public health facilities including mobile unit services each with recorded geographic coordinates (longitude/latitude), from 51 local municipalities including the metros, obtained from the KZN Provincial Department of Health DHIS for the reporting period 2015/16. Twenty three facilities did not have positivity rates data and were excluded. The data for the 2015/16 reporting period was the most complete to undertake the spatial analysis, as at the time the DHIS team was updating the DHIS data collection forms for the following reporting years. The mobile facilities (183), though in some cases were located in close vicinity to the main hospitals or clinics, had their own unique 1st test cases and hence were treated as unique data points in the analysis. In terms of age, the DHIS data records three age categories, 0–14, 15–49, and 50 years and above, and data is not disaggregated by sex, which is one of the key limitations of the data. For this study the details of included data indicator, level of aggregation, data sources, and facilities inclusion criteria are provided as Supplementary Material. The HIV positivity indicator represents the proportion of clients 15–49 years on whom an HIV test was done who tested positive for the first time at public health facilities as aggregated annually in the DHIS. The following calculation was applied to generate HIV positivity at facility level:

$$\begin{array}{l} HIV\;positivity\;at\;facility\;\left(15-49\right)\\ =\frac{HIV\;1st\;test\;HIV\;positive\;\left(15-49\right)\;}{\;Total\;HIV\;1st\;test\;\left(15-49\right)}*100 \end{array}$$

The data does not include self-reported positives which is noted as a limitation in using HIV positivity rate as a proxy for prevalence.

#### Gridded-High Resolution Population Data

For the population data we used the Worldpop gridded high resolution (100 m grid cell) population data from https://www.worldpop.org, WorldPop Data Repository (8). The gridded population data is based on well-tested models incorporating population density, land cover, and urban/rural disaggregation. In addition, the data are validated and calibrated using national census data. The WorldPop data has also been used widely in modeling disease burdens across the world including generation of the HIVE-Map model supported by UNAIDS (9). There is generally good agreement in results derived from different methods including the HIVE-Map, disaggregation of Spectrum projections, and small-area estimation. This gives us confidence in using the World population data in this study to generate a raster surface of the population aged 15–49 years at 100 m resolution.

## Methods

All analyses were performed using the Arc-GIS10.0 Ver 2.18.24 (10) software which is also available in the KZN Department of Health data source platform making it easier for decision makers to use the tool with updated routine data. The analysis can also be done with open sources geographical information system (QGIS) (11). To establish the high HIV burden areas, the study provides a step by step approach that can be easily replicated by the local decision makers with updated data.

### Step 1

Combine 2015 South Africa high resolution (100 m) gridded population and 2015 South Africa mid-year population age-structure data estimates, to generate a raster surface of the number of people aged 15–49 years old in KZN at 100 m resolution *(population15–49)*.

### Step 2

Use the health facilities HIV positivity rates among 15–49 years old to conduct hot spots analysis as a first step to identify areas that are statistically significant hotpots (i.e., locations of health facilities with a significantly high number of HIV positive cases) and vis-versa (cold spots) (12). Then apply the inverse weighted distance [IDW, (12)] interpolation method on health facility positivity rate data for 15–49 years olds individuals to generate unadjusted HIV positivity raster surface at 100 m resolution (*positivity15to49_unadjusted)*. The IDW is a deterministic technique; exact interpolator, which means theoretically we produced the exact value given at a sample point. The IDW approach generates a continuous smooth HIV positivity surface from point based data by calculating the parameter values at an unmeasured point using a distance-weighted average of data points. The IDW uses only the values of the known sample points to estimate unknown points of interest. For the study we used 100 data points and the squared distance to reduce the number of calculations making the IDW approach effective for reducing the amount of computation needed to produce an estimate. Selection of the 100 data points for estimation around unknown locations was done using the adaptive approach, which is more relevant in health in order to get a closer match of the spatial distribution of the population and thus reduce the smoothing of information. Also, an adaptive bandwidth of equal number of points makes it possible to achieve a smoothing effect that adapts to the high irregularity of spatial distribution among the facility locations, selecting the facility locations according to the observed population distribution. Surface generated is more accurate for densely populated areas (as more observations are available) and strongly smoothed in sparsely surveyed areas.

### Step 3

Use ArcGIS spatial analyst map algebra to combine the gridded surface map for 15–49 years population group (*population15-49)* and HIV positivity surface map (*positivity15to49_unadjusted)* to get the unweighted surface map of PLHIV (*plhiv15-49_unadjusted*), which is then proportionately rescaled to the Provincial and National South Africa‘s published HIV estimates to generate the adjusted surface of 15–49 years olds PLHIV at 100 m resolution. For local decision making, results are then aggregated to required administrative units (i.e., districts, municipalities) by adding pixels of the surfaces to find the total number of people PLHIV per unit. Aggregate total population per unit can also similarly be calculated from the population surface map. Further, dividing PLHIV per unit by total population per unit provides adjusted HIV positivity surface per unit for the 15–49 years old population group. We also estimated an error surface per administrative unit to show the quality of the estimate. To assess uncertainty of the estimates at administrative unit, we adopted Larmarange and Bendaud approach (13), and for each administrative unit compared the number of observations(obs) in each unit and the number of points (N) used in the spatial estimation of the positivity surface, and define estimates as “uncertain” if 0 < obs < *N*/2 (estimates are mostly based on observations from neighboring units), “moderately good” if *N*/2 < obs < *N* (estimates are partially based on observations from the same unit), and “good” obs is at least *N* (estimates are based on observations from the same unit).

Other methods that have been used to generate local level estimates include kernel density estimation with adaptive bandwidths, Bayesian modeling and small area estimation (14–19). All these methods have been mostly applied using national household HIV surveys and the Antenatal Care Sentinel Surveillance datasets rather than routinely corrected health facility data. Our study demonstrates the use of routine data and spatial interpolation methods in estimating high disease burden areas and can be rolled out to other regions in South Africa and also to other LMIC countries with available routine health data.

## Results

**Figure 1:**
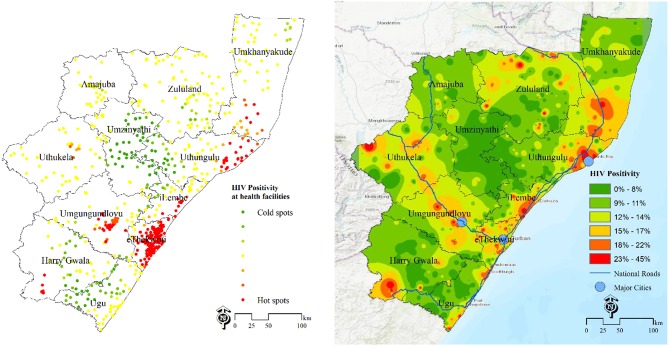
Estimation of HIV disease burden based on positivity rates data DHIS 2015/16. **Left**-HIV positivity at health facilities, 2015/16; **Right**-population unweighted HIV positivity surface at 100 m resolution.

Figure 1, left shows the “hotpots” with high HIV positivity rates surrounded by other facilities with high values. The cold spots (green) have low HIV positivity and are surrounded by other facilities with low values. This map identifies locations of health facilities with a significantly high number of HIV positive cases. The “hotspots” are clearly identified in eThekwini, uMgungundlovu, iLembe, uThungulu and uMkhanyakude. From the map of interpolated HIV positivity surface (Figure 1, right), the main “hotspots” areas are in eThekwini, iLembe and uMngundgudlovu. Overlaying the major cities and national routes show high burden areas (hotspots) around major cities and routes (Figure 1, right).

**Figure 2:**
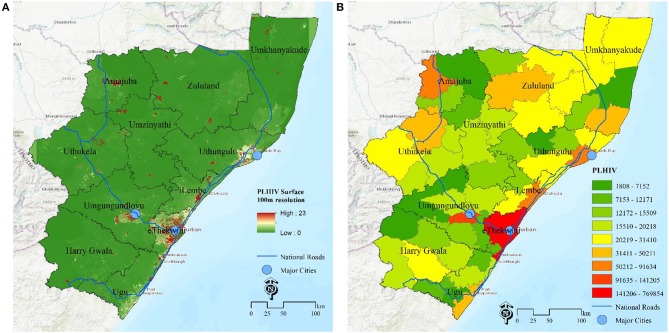
Estimation of HIV disease burden based on number of PLHIV. **(A)** PLHIV at 100 m gird resolution, **(B)** PLHIV at municipality, KwaZulu Natal, South Africa.

An additional approach of estimating the disease burden is the surface map of the number of PLHIV at grid level (Figure 2A) and estimates of number of PLHIV aggregated at selected administrative unit (Municipality, Figure 2B) for decision making Based on these maps, they major hotspots (red) are eThekwini municipality and a municipality in uMgungundlovu identified as carrying the highest HIV disease burden with high number of PLHIV. The rest of municipalities are yellow-green. Due to the effect of the area population size, areas with high population, mostly the major urban areas, have high absolute numbers of PLHIV, over areas with low population size, even when they both have same HIV positivity rates. All these maps shows complementary pictures of the burden of HIV in KwaZulu Natal.

**Figure 3:**
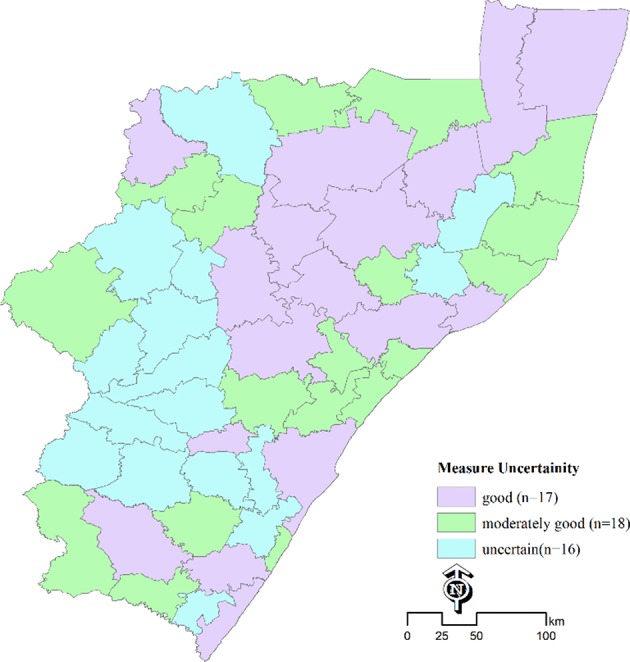
Measure of uncertainty for estimates of PLHIV at municipality.

Figure 3 shows estimates of measure of uncertainty at municipality level, with estimates being uncertain in ~16 out of 51 municipalities. These were mostly the areas where estimates were based on samples data or observations from neighboring areas.

## Discussion

In a constrained funding environment, identifying areas of high disease burden allows decision-makers to target resources for the greatest impact. We provide a step-by-step approach that can allow local decision makers to use routinely updated facilities data autonomously to reproduce estimates at subnational levels to guide efficient allocation of resources. The study results have identified municipalities with high HIV disease burden using public health routine facility data for 15–49 years old. The high burden areas are around the major urban centres including municipalities in UMgungundlovu, eThekwini; places near major road networks; and along the coastal belt, observed by overlaying layers of road networks and cities. The findings parallel those of recent studies (1, 20, 21) that have provide subnational HIV prevalence estimates, but using survey data. Wanyeki et al. (20) study used routine facility-level Prevention of Mother to Child Transmission (PMTCT) data to indicate high burden areas at district level, showing that the HIV burden is concentrated in main urban centers similar to findings in this study. Dwyer-Lindgren et al. (1) study explored within-country variation at a 5 × 5-km resolution revealing substantial within-country variation in the prevalence of HIV among adults (aged 15–49 years) across sub-Saharan Africa 2000–2017, and similarly Gutreuter et al. (21), using Antenatal surveillance survey (ANC) as a covariate provided substantially improved precision in many district-level estimates of HIV prevalence in the general population using national survey data.

Our study shows different but complementary pattern of disease burden based on HIV positivity surface and the PLHIV surface map which can be attributed to large effect of area population size on numbers of PLHIV. Gutreuter made similar observations on his work on district prevalence estimates. The concentration of population in urban centers including eThekwini means a high number of PLHIV around the urban centers, with lesser number of PLHIV in the rural and remote areas. Accessibility also mean we have a high number of people using facilities selected areas near major access routes. Similarly, a study by Tanser et al. (19) showed that HIV is localized in areas neighboring major routes. All these studies used sero-prevalence and/or ANC to map HIV prevalence at subnational levels. The current study focused on modeling and mapping geographical areas with high HIV disease burden using routine facility data. With ability to delineate high disease burden areas, with readily available tables for different administrative levels, the local decision makers can then generate the associated risk profiles to guide decision making.

Few peer reviewed research articles have used routine facility level data to model the disease burden for decision making. The method applied in this study is readily applicable in other settings including other low and medium income countries (LMIC), but will involve working together with the data custodians in those countries. Working with data custodians will also help to improve the data systems by identifying data gaps and improving on tools for data collection providing rich data for better analysis and local planning. The analysis did not take into account contribution of associated risks including male urethral syndrome (MUS), other sexually transmitted infections, and teenage pregnancies. Further research will entail application of spatial multi-criteria decision making (22) approaches for incorporating risk factors in a bid to further define potential high-risk areas. It is also important to explore data on HIV service coverage as tremendous value can be derived from linking health facility data to community research datasets to generate population-level estimates of coverage with HIV services, which is at the heart of the South Africa National Strategic Plan (NSP) strategy to “focus for impact” (23).

## Study Limitations

There are limits to using available routine health data for spatial modeling. First, data on HIV testing are routinely collected in public health care facilities using paper-based registers corresponding to distinct HIV care and treatment service spectrum. The data are aggregated per health facility and fed DHIS. The manual data entry has the potential of introducing data capture errors in the system. A role out of web-based systems of data capture entry, currently underway in some regions will help eliminate some of these problems as the users can directly capture the information at the source. But this will also mean availability of good internet connectivity.

Second, routine data has the inherent limitation with respect to maintaining and accurately recording unique identifiers that can link patients across the different facilities. This also poses challenges when compiling aggregate data due to possible double counting for patients who visit multiple facilities. For this study, we only used first test cases to avoid double counting of individuals. An improvement to the overall DHIS system should include the use unique identification to help track individuals across the health facilities making them easily identifiable, while maintaining individuals' confidentiality. In addition, the fact that individuals tested at health facilities self-select, means that those who do not access services at public health facilities are not part of the analysis.

Third, the routine health facility testing data does not include self-report cases, which despite the bias associated with self-reported measurements can be used to complement the reported HIV positivity rates as a good proxy measure of HIV prevalence (23). The routine health facility indicators data age breakdown is limited to only three age categories, 0–14, 15–49, and 50 plus years; does not capture sexual risk data; and sex and gender breakdown data. This means for example, no key population specific data can be segregated from available routine datasets. This is a serious limitation in available routine data sets. Excluded from the analysis include data from prisons and data on preferences for service utilization since they are not part of the routinely collected data. We also focused on all adults 15–49 years excluding antenatal data.

Fourth, in terms of the spatial interpolation approach, assumptions made including (i) taking the age-structure of population to be the same across all administrative units (i.e., the spatial distribution of individuals 15–49 years is the same as the spatial distribution of the overall population as estimated by Worldpop); (ii) assuming available data are up to date and data quality rules are applied uniformly and consistently across the health facilities; (iii) assuming all populations have equal HIV risk, which is a concern as this could mask key and vulnerable populations; and finally (iv) assuming that neighborhood areas tend to have similar HIV positivity [Tobler's first law (TFL) of geography (24)] also need to be noted. Additionally, because the mobile clinic data is geographically mapped to the same coordinate points of its parent facility (point where mobile is working from) and not mapped according to service delivery routes, this is likely to skew the outcome when data is projected geospatially.

The study is based on one time point and future analysis should include more time periods to establish trends.

## Data Availability Statement

The datasets analyzed for this study are from the Health District Health Information system owned by the Kwa-Zulu-Natal department. Data can be obtained by signing a Data user agreement form with the Department of Health that stipulates that the use of datasets in research communication, scholarly papers, journals and the like is encouraged with acknowledgment of the Kwa-Zulu Department of Health as the data source.

## Ethics Statement

The study received full ethics approval from the UMgungundlovu Health Ethics Research Board (Ref: UHERB 003/2019) which is registered with the South Africa National Health Research Ethics Council (NHREC) under reference REC-051010-026.

## Author Contributions

NW and IN conceptualized the validation methods, and wrote the first draft. EM, CS, and TN provided access to the data. NW incorporated all comments and generated final draft. All authors reviewed and approved the final manuscript for submission.

### Conflict of Interest

The authors declare that the research was conducted in the absence of any commercial or financial relationships that could be construed as a potential conflict of interest.
