# Supplementary material for: The Arts and Tools for Using Routine Health Data to Establish HIV High Burden Areas: The Pilot Case of KwaZulu-Natal South Africa
Source: Front Public Health. 2019 Nov 12;7:335. doi: 10.3389/fpubh.2019.00335 (PMC6861206; doi:10.3389/fpubh.2019.00335)
Supplement: Supplementary file 1 [file Data_Sheet_1.docx]

The indicators, level of aggregation and data source. Also include the facilities inclusion criteria

**Table 1: Indicators, Level of Aggregation and Data Source Data**

| DATA | **Key Indicators/Variables** | **Date** | **Level of Aggregation** | **Source** |
| --- | --- | --- | --- | --- |
| **Public health care facility routine data from DHIS** | Annual HIV positivity among adults 15-49 years clients tested at health facilities | 2015/16 financial year | Clinic | DHIS |
| **Administrative boundaries** | ^+^Catchment areas | 2011 | Province, district, municipality | StatSSA, 2011 census/population data |
| **Gridded population** | South Africa gridded population | 2015 | 100 X100 m grid cell | <https://www.worldpop.org> (World gridded population data) |
| **Mid-year population estimates** | Population age structure (15-49 years old ) | 2015 | National, Province | StatSSA. Mid-year population estimates, 2015 |
| **Master facility list** | Latitude, longitude of health facilities | 2014 | Clinic | DHIS |
| ^*^Collected from monthly facilities data collection forms (Appendix A and B), which are then entered in the electronic DHIS database.  ^+^ Catchment Areas- boundary files from 2011 census. Results can be aggregated to any decision making geographical boundary level (i.e. districts, municipalities). | | | | |

HIV prevalence amongst client tested 15-49 years rate

**Table: Inclusion and Exclusion Criteria for health facilities**

| **Inclusions** | Public health facilities, DHIS reporting on clients 15-49 years tested for HIV for reporting financial year 2015/2016 | | **887** |
| --- | --- | --- | --- |
| **Exclusions** | Health facilities with missing/ not reported HIV positivity rates amongst clients 15-49 years | | 23 |
| **Total facilities included for analysis** | | **865** | |


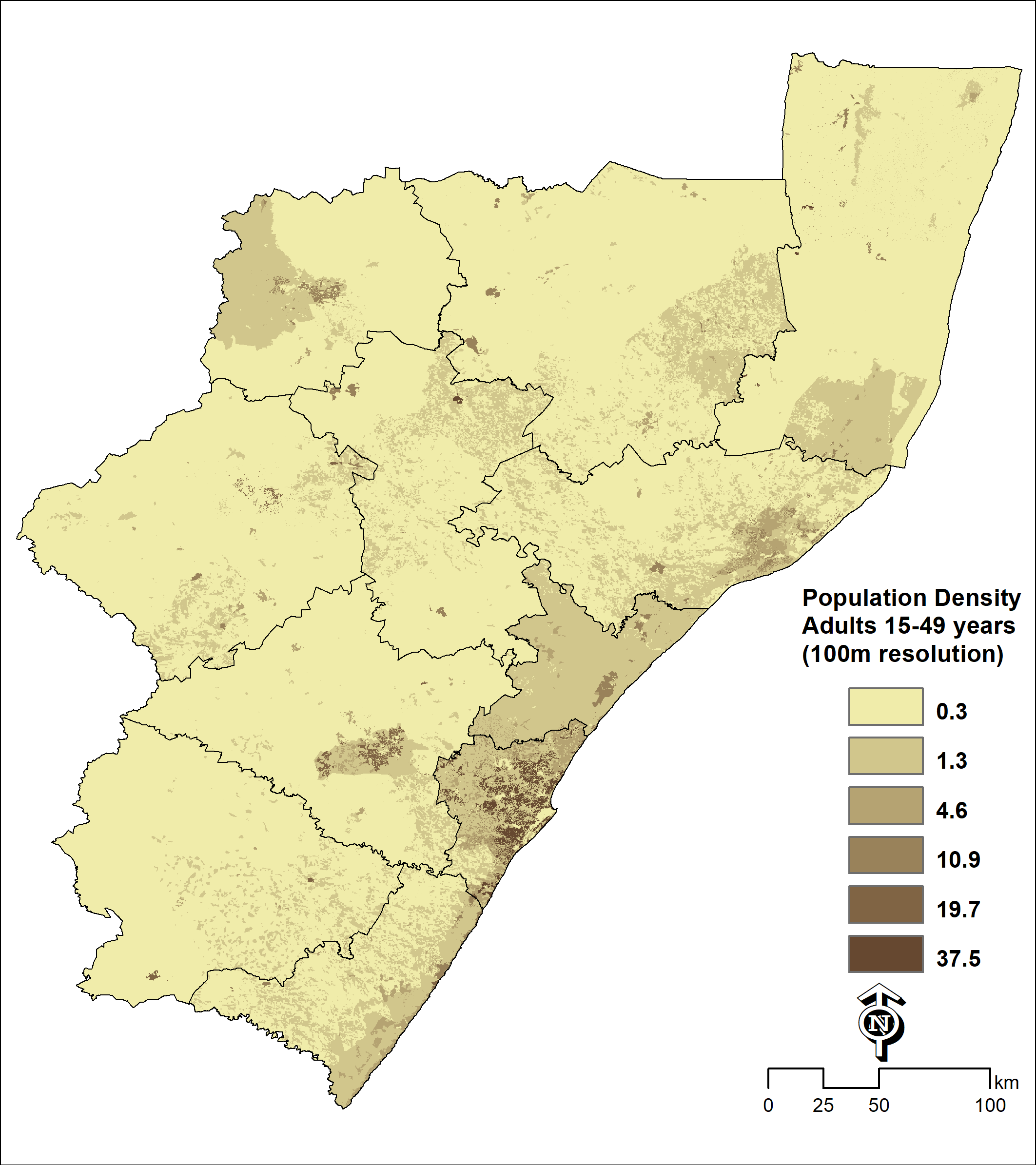


Figure 1 Population Density of adults 15-49 years at 100m resolution
